# G×G×E for Lifespan in *Drosophila*: Mitochondrial, Nuclear, and Dietary Interactions that Modify Longevity

**DOI:** 10.1371/journal.pgen.1004354

**Published:** 2014-05-15

**Authors:** Chen-Tseh Zhu, Paul Ingelmo, David M. Rand

**Affiliations:** Department of Ecology and Evolutionary Biology, Brown University, Providence, Rhode Island, United States of America; Max Planck Institute for Biology of Ageing, Germany

## Abstract

Dietary restriction (DR) is the most consistent means of extending longevity in a wide range of organisms. A growing body of literature indicates that mitochondria play an important role in longevity extension by DR, but the impact of mitochondrial genotypes on the DR process have received little attention. Mitochondrial function requires proper integration of gene products from their own genomes (mtDNA) and the nuclear genome as well as the metabolic state of the cell, which is heavily influenced by diet. These three-way mitochondrial-nuclear-dietary interactions influence cellular and organismal functions that affect fitness, aging, and disease in nature. To examine these interactions in the context of longevity, we generated 18 “mito-nuclear” genotypes by placing mtDNA from strains of *Drosophila melanogaster* and *D. simulans* onto controlled nuclear backgrounds of *D. melanogaster* (*Oregon-R*, *w* ^1118^, *SIR2* overexpression and control) and quantified the lifespan of each mitonuclear genotype on five different sugar:yeast diets spanning a range of caloric and dietary restriction (CR and DR). Using mixed effect models to quantify main and interaction effects, we uncovered strong mitochondrial-diet, mitochondrial-nuclear, and nuclear-diet interaction effects, in addition to three-way interactions. Survival analyses demonstrate that interaction effects can be more important than individual genetic or dietary effects on longevity. Overexpression of *SIR2* reduces lifespan variation among different mitochondrial genotypes and further dampens the response of lifespan to CR but not to DR, suggesting that response to these two diets involve different underlying mechanisms. Overall the results reveal that mitochondrial-nuclear genetic interactions play important roles in modulating *Drosophila* lifespan and these epistatic interactions are further modified by diet. More generally, these findings illustrate that gene-by-gene and gene-by-environment interactions are not simply modifiers of key factors affecting longevity, but these interactions themselves are the very factors that underlie important variation in this trait.

## Author Summary

It is widely recognized that mitochondrial function plays an important role in longevity and healthy aging. Considerable attention has been focused on the extension of longevity by caloric or dietary restriction and mutations that alter this process, and these interventions commonly are associated with shifts in mitochondrial function. While the genetic bases of these effects are the focus of much interest, relatively little effort has been directed at understanding the role that mitochondrial DNA (mtDNA) polymorphisms play in the diet restriction response. This work presents a comprehensive effort to quantify the effects of mtDNA variants, nuclear genetic variants and dietary manipulations on longevity in *Drosophila*, with a focus on testing for the importance of the interactions among these factors. We found that mitochondrial genotypes can have significant effects on longevity and the diet restriction response but these effects are highly dependent on nuclear genetic (G) background and the specific diet environment (E). For example, a mitochondrial haplotype that shortens lifespan in one nuclear background or diet regime shows no such effect when the genetic background or diet regime is changed. Our experiments indicate that identifying individual mitochondrial, nuclear or dietary effects on longevity is unlikely to provide general results without quantifying the prevalent mitochondrial × nuclear × diet (G×G×E) interactions.

## Introduction

The extension of longevity by dietary or caloric restriction (DR or CR) has been demonstrated in a wide range of organisms, suggesting evolutionarily conserved pathways that regulate this response [1], [2], [3], [4], [5]. However, dissecting the genetic and cellular mechanisms of DR remains a great challenge. Several pathways have been identified that mediate the DR response such as the insulin [6], [7], mTOR [8], [9], [10], AMPK [11], [12], [13], [14] and sirtuin pathways [15], [16], [17], [18]. These pathways intersect through downstream targets and involve mechanisms of regulation and feedback. Thus, the control of DR involves a network of pathways rather than any one critical pathway, which underlies the challenge of identifying singular genetic and biochemical mechanisms for the DR response. While efforts to identify single genes, pathways or nutrients that extend longevity have been productive, studies that focus on the interaction among relevant factors have received relatively little attention [19]. Longevity, and its extension by genetic or dietary interventions, are complex phenotypes, and it is increasingly apparent that gene-by-gene (G×G) and gene-by-environment (G×E) interactions are fundamental components of these kinds of traits [20], [21]. Given the complexity of the genetic, nutritional and physical environments in which most organisms live, including humans, explicit studies of the role of these interaction effects are critically important to determine the generality of single factor analyses.

The mitochondrion has received increasing attention as a nexus for regulation of the longevity extending effects associated with DR [10], [22], [23], [24], [25]. Mitochondria generate critical energy stores in the form of ATP and NADH that can promote cellular maintenance and longevity, but also generate reactive oxygen species (ROS) that can cause cellular damage and senescence. As a hub for input from multiple pathways affecting longevity, mitochondria provide a compelling target for studies seeking to understand gene-by-gene and gene-by-environment effects in aging. Mitochondrial function and biogenesis are dependent on genes encoded in both the mtDNA and the nuclear chromosomes. Animal mtDNA encodes 13 protein coding subunits of oxidative phosphorylation complexes of the electron transport chain (ETC) with more than 70 remaining subunits encoded by nuclear genes and imported in to the mitochondrion [26], [27]. mtDNA also encodes a minimal set of RNAs (2 rRNAs and 22 tRNAs) that comprise the translation machinery inside the mitochondrion, with the remaining protein components, such as ribosomal proteins and tRNA synthetases, encoded by nuclear genes and imported into the organelle. Thus, mitochondrial function depends critically on the coordination of mitochondrial and nuclear encoded components and this co-dependence provides the basis of mitochondrial-nuclear interactions (hereafter mitonuclear interactions), which is one mode of gene-by-gene interaction, or epistasis, that may affect fitness, disease and aging [28], [29], [30].

Specific genes have been identified that play important roles in modifying mitochondrial function in response to DR. The TOR pathway regulates nutrient sensing and protein translation [31], [32], and 4E-BP regulates differential translation of mRNAs encoding proteins targeted for mitochondria vs. cytosolic function [10]. The PGC-1α family of transcriptional co-activators also plays a critical role in regulating mitochondrial biogenesis with systemic and tissue-specific effects on longevity [22], [33]. Altered expression of specific subunits of electron transport chain (ETC) complexes can reduce the efficacy of longevity extension by DR [10], [34], suggesting that the coordination of nuclear and mtDNA-encoded components of mitochondrial function are important in mediating a proper response to DR. Genetic analyses of longevity extension by DR are examples of the more general problem of genotype-by-environment interaction, as the question concerns the identification of genes or alleles that generate a novel phenotype (longer life) in a novel environment (reduced protein or calories). Thus, mitochondrial regulation of DR or CR not only requires the complex mitonuclear interactions, but also involves the impact of the dietary environment on this gene-by-gene interaction. Collectively, this network can be summarized as a three-way interaction between mitochondrial genotype, nuclear genotype and dietary environment denoted as G×G×E in quantitative and ecological genetics.

**Figure 1:**
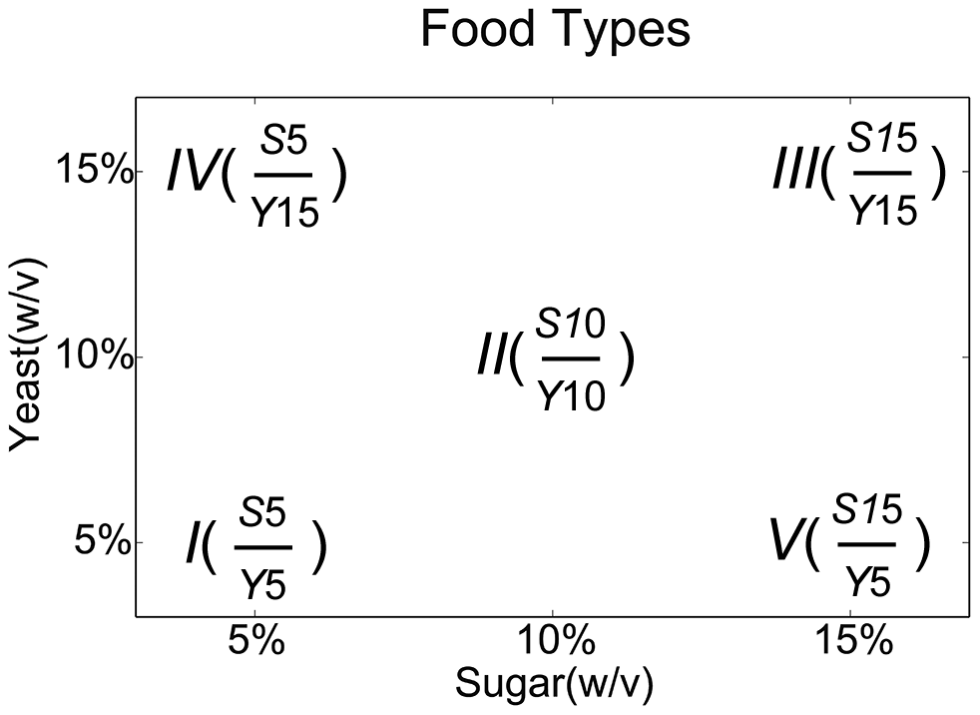
Diagram of diets used in this study. The horizontal axis shows the sugar concentration and the vertical axis shows yeast concentration, both as weight/volume. The Roman numerals are the short-hand used in the text and figures, and the numbers in parentheses show the ratios of sugar/yeast. The diets I-II-III span a diet concentration axis reflecting caloric restriction(CR); the diets IV-II-V span a diet composition axis reflecting diet restriction (DR).

In this study we explicitly unite all three of these issues: mtDNA variation, nuclear gene variation and dietary variation to test hypotheses about the generality of single-factor effects on longevity under DR and CR. By employing a 5-diet design, we attempt to make the distinction between CR and DR. In caloric restriction (CR) only the caloric content of the diet is reduced while the relative composition of macro-nutrients is kept untouched. In dietary restriction (DR), one focal nutrient of the diet, such as the proportion of protein or sugar, is restricted with the caloric content kept constant (see Figure 1). We address the questions of main effects and interaction effects directly using *Drosophila* genetic tools to jointly manipulate mitochondrial and nuclear gene mutations, and examine these individual and combined effects on lifespan in response to dietary alterations. We demonstrate that mtDNA genotype alters the dietary effect on lifespan in each of two commonly used nuclear genetic backgrounds of *D. melanogaster*. We also find that mtDNA haplotypes can alter the effect of *SIR2* overexpression on life span in a diet dependent manner. These results demonstrate the three-way mito-nuclear-diet interaction as an important factor in shaping longevity outcomes. These results imply that an accurate assessment of mitochondrial factors cannot be made without the context of nuclear genetic background and dietary regime, each of which has been implicated as a single important factor influencing longevity.

## Results

### Caloric and dietary restriction have genotype-specific effects on longevity

**Figure 2:**
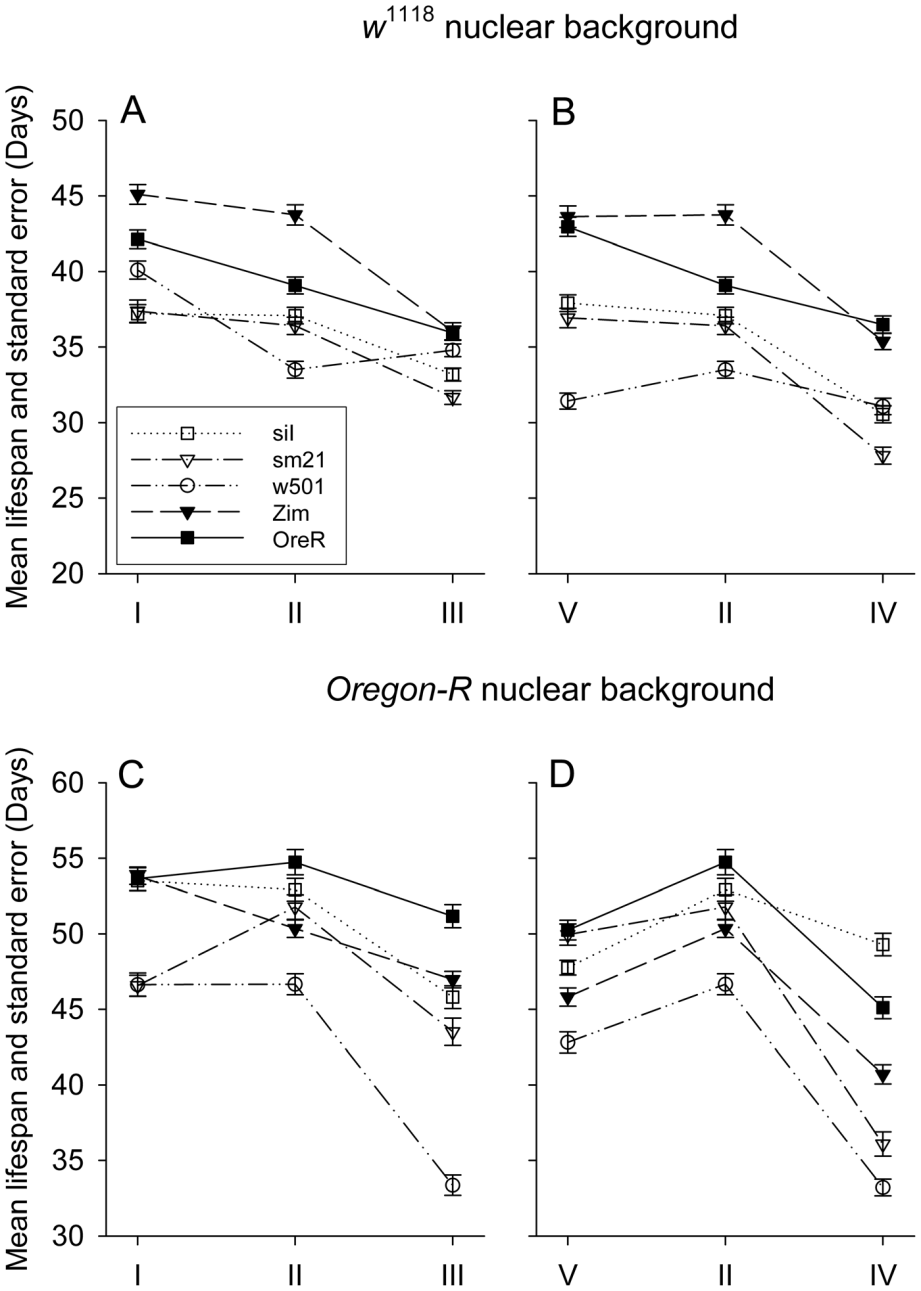
Mean lifespan response to diet alterations of different mitotypes in *OreR* (A and B panels) and *w* ^1118^ (C and D panels) nuclear backgrounds. Food concentration gradient is presented in the left panels (A and C) and food composition gradients in the right panels (B and D). Error bars represent the standard error of the mean. Mitotypes are presented with different markers and line styles. *D. simulans* mtDNAs have open symbols: siI, square marker with dot line; sm21, triangle marker with dot-dash line; w501, circle marker with double-dot-dash line; *D. melanogaster* mtDNAs have filled symbols: Zim, filled triangle with dash line; and OreR, filled square with solid line. Different genetic backgrounds, *OreR* and *w* ^1118^, are shown in lower and upper panels respectively.

For each of two axes of dietary manipulation, both the concentration axis and the composition axis (see Figure 1), there was a robust lifespan response in each of two standard *D. melanogaster* strains, *w* ^1118^ and *OreR* (Figure 2 and Table 1). The concentration axis is made of food type I, II and III (Figure 2A and C). There is a clear pattern that mean lifespan declines with food concentration. Strain *w* ^1118^ is shorter-lived than *OreR* (comparing Figure 2A, B to Fig. 2C, D), which also agrees with earlier work where *w* ^1118^ is generally found to be a highly fecund but short-lived strain [35], [36].

**Table 1:** Summary of hazard ratios on different diets in *w* ^1118^ and *OreR* nuclear genetic backgrounds.

	*log*(hazard ratio)	hazard ratio	S.E.M.	*z*	*P*
*w* ^1118^ nuclear background					
Diet I	−0.2290	0.7953	0.0416	−5.51	3.59×10^−8^
Diet III	0.6648	1.9442	0.0423	15.70	1.51×10^−55^
Diet IV	0.6935	2.0007	0.0424	16.36	3.69×10^−60^
Diet V	−0.2120	0.8090	0.0420	−5.05	4.42×10^−8^
*OreR* nuclear background					
Diet I	0.1123	1.1188	0.0416	2.70	0.0069
Diet III	0.5193	1.6809	0.0420	12.38	3.35×10^−35^
Diet IV	0.7603	2.1390	0.0429	17.73	2.46×10^−70^
Diet V	0.4113	1.5088	0.0416	9.88	5.08×10^−23^

CR and DR experiments use type II diet as a reference. Negative *log*(hazard ratio) values indicate that the tested diet causes a lower risk of death, and positive values indicate a higher risk of death, respectively, than type II. See Figure 1 for diets, and Materials and Methods for details.

Food type V, II and IV make up a composition axis, where the sugar:yeast ratio is 3∶1, 1∶1 and 1∶3, respectively. In both nuclear genetic backgrounds, it is apparent that the low protein and the balanced diets results in long lifespan, with the high protein diet resulting in reduced lifespan (Figure 2B, D). Pooling data crosses all mitotypes and just focusing on the dietary affect, the estimates of hazard ratio of different diets are summarized in Table 1. It shows that compared to the median level diet (type II), the high yeast diet (type IV) and high caloric diet (type III) roughly double the chance of death (see Table 1, column ‘hazard ratio’: for *w* ^1118^ the risks of death for diet types III and IV are 1.9442 and 2.0007 relative to type II; for *OreR* these values are 1.6809 and 2.1390).

In *Drosophila* studies, yeast is usually the only protein source, and increase in yeast concentration leads to high fecundity and short lifespan [36]. It has been further demonstrated that rather than yeast concentration alone, sugar/yeast ratio is what leads to the longevity and reproductive changes [37], [38]. Here we have a high sugar food, a high yeast food and a balanced food with their caloric content roughly being equal. While altering either the diet composition or concentration significantly affects lifespan (*P*<0.001 for both strains), different nuclear genotypes appear to have different optimal sugar/yeast ratios that result in maximum longevity (compare *w* ^1118^ to *OreR* in Figure 2B and D; Note that this figure presents data for multiple mtDNA types, described in the following section).

### mtDNA effects are restricted to individual haplotypes, not species-level divergence

To examine the effects of mtDNA genotype on longevity, we generated 18 ‘mito-nuclear’ genotypes by placing mtDNA from strains of *Drosophila melanogaster* and *D. simulans* onto controlled nuclear backgrounds of *D. melanogaster* (*Oregon-R*, *w* ^1118^, *SIR2* overexpression and control; see Methods). In each diet regime, on each nuclear background, the individual mitotypes show significant variation in longevity (*P*<2×10^−16^; Table 2, effect of mito; note: ‘nucleartype’  =  nuclear genetic background; ‘mitotype’  =  individual mtDNA genotype). Mitochondrial effects are largely due to variation among individual mitotypes while species-level mtDNA sequence divergence surprisingly contributes very little (compare *D. simulans* mitoypes siI, sm21, w501 to *D. melanogaster* mitotypes OreR and Zim; Figure 2 A–D). Although in some cases it appears *D. simulans* mitotypes are shorter lived than *D. melanogaster* mitotypes on specific diets (*w* ^1118^ background under CR, Figure 2A), this species divergence effect is not significant across all diets given the magnitude of within-species variation observed. Comparing a model where individual mitotypes are treated separately to a model where mtDNA variation is treated as a species divergence effect with individual mitotypes nested within species divergence, the latter model is not significantly better in the *OreR* nuclear background (table 2, *χ^2^* = 0.1113, n.s), indicating the lack of support for an mtDNA species-divergence effect. In the *w* ^1118^ nuclear background, the species effect (*χ^2^* = 3.7314, *P* = 0.053) is marginally significant. While the support for a species-level effect of mtDNA on longevity is again not strong, this effect does appear to be stronger in *w* ^1118^ than in the *OR* nuclear background. The variation among mitotypes for longevity is, however, very significant in each nuclear background (table 2, *χ^2^* = 156.07 for *OreR* and *χ^2^* = 556.76 for *w* ^1118^, d.f. = 2, *P*<0.001 for both).

**Table 2:** Mixed effect Cox proportional hazard models for survivorship in *w* ^1118^ and *OreR* CR/DR experiments.

		Nuclear background
		*Oregon-R*		*w^1118^*		
Effect	d.f.	*χ^2^*	*P*	*χ^2^*	*P*	Compare Models:
diet	4	212.56	7.48×10^−45^***	403.26	5.49×10^−86^***	
mito	1	156.07	8.17×10^−36^***	556.76	4.26×10^−123^***	Model 0 & 1
species	1	0.1113	0.7387	3.7314	0.0534 †	Model 1 & 2
dietary effects:						
depends on mito	2	149.68	3.14×10^−33^ ***	45.813	1.13×10^−10^***	Model 2 & 3
depends on mito|species	1	0.0129	0.9097	0.0001	0.9966	Model 3 & 4
replicate	1	10.659	0.0011 **	0.0022	0.9629	Model 4 & 5

Significance codes: 0 ‘***’ 0.001 ‘**’ 0.01 ‘*’ 0.05 ‘†’.

Model 0: diet. *(Note 1).*

Model 1: diet + mito.

Model 2: diet + mito|_speices_ + species *(Note 2).*

Model 3: diet + diet|_mito_ + mito|_speices_ + species *(Note 3).*

Model 4: diet + diet|(mito|_speices_ + species) + mito|_speices_ + species *(Note 4).*

Model 5: diet + diet|(mito|_speices_ + species) + mito|_speices_ + species+ replicate.

Note 1: fixed effect, such as diet, is shown with underline.

Note 2: mitochondrial effect, as a random effect, nested within species; ‘species’ and ‘line nested within species’ are treated as categorical variables and not scaled by sequence divergence.

Note 3: additional dietary effect modeled as an effect nested within mitochondrial effect.

Note 4: additional dietary effect modeled as an effect nested within mitochondria and species effects.

Dietary effects are modulated by mitochondrial genotypes and this genotype-by-environment interaction (G×E; technically a mitotype-by-environment (M×E) interaction) is also a big contributor to survivorship. We found dietary effects to be variable in different mitotypes (Table 2, *χ^2^* = 149.68 in the *OreR* nucleartype background and *χ^2^* = 45.813 in the *w* ^1118^ nucleartype background, d.f. = 1, both *P*<0.001). However, the same is not true for species mtDNA divergence: dietary effect is virtually not altered by the species-level mtDNA divergence effect on either nuclear background (table 2, *χ^2^* = 0.0129 for *OreR* and *χ^2^* = 0.0001 for *w* ^1118^, d.f. = 1, both insignificant). The use of independent contrast approaches, or scaling for mtDNA branch length does not alter the outcome of these analyses (data not shown). Together these results indicate that mtDNA polymorphisms have a more pronounced effect on longevity than the species-level mtDNA divergence of ∼100 amino acids and a total of 571 fixed nucleotide substitutions between the mtDNAs of these two species.

### A two-nucleotide mito-nuclear epistasis shortens lifespan

In the statistical analyses for the previous section, mitotype w501 was excluded from the results reported in Table 2 as it is known to have a strong negative epistatic interaction with the *Oregon-R* nuclear background [29]. The *D. simulans* w501 and sm21 mtDNAs differ from each other by six mutations, 5 of which are synonymous or indels in non-coding DNA, and the only putative functional mutation is in the anticodon stem of the mitochondrial tRNA^Tyr^ gene. Further genetic mapping shows that the w501 mtDNA interacts negatively with a mutation in the nuclear-encoded gene for mitochondrial-tRNA^Tyr^ synthetase [29]. This gene is polymorphic in *D. melanogaster*: the *OreR* genetic background carries the mutant allele and *w* ^1118^ carries the wildtype allele for mitochondrial tRNA^Tyr^ synthetase on chromosome 2. Transgenic analyses of this single nucleotide show that it is responsible for the negative interaction with the point mutation in the tRNA^Tyr^ gene of w501 mtDNA [29].

Our longevity results show that the w501 mitotype is generally shorter-lived than sm21 in *OreR* but not in *w* ^1118^, which carries the wild type allele of the nuclear-encoded tRNA^Tyr^ synthetase (Figure 2 a–d). Therefore, the lifespan difference caused by the mt-tRNA^Tyr^ mutation between w501 and sm21 appears to be strongly dependent on nuclear background, as is true for several other phenotypes [29], [39]. This empirical pattern has very strong statistical support. The w501 mtDNA -by-nuclear term is highly significant (*χ^2^* = 132.98 on 1 degrees of freedom, *P*<0.001). It can be further determined that in the *OreR* genetic background, the sm21 mitotype has about 53.8% risk of death relative to the w501 mitotype, which is highly significantly lower (Table 3, *P*<0.001). In contrast, in the *w* ^1118^ genetic background, the risk of death in sm21 and w501 mitotypes are not significantly different (Table 3, *P* = 0.952).

**Table 3:** Summary of hazard ratios of background effects and w501 mitotype effects in *OreR* and *w* ^1118^ nuclear backgrounds using OreR as the reference nuclear genotype.

	*log*(hazard ratio)	hazard ratio	S.E.M.	*z*	*P*
Between *OreR* and *w* ^1118^ backgrounds	0.7306	2.0762	0.0168	43.58	0.0
Between w501 and sm21 mitotypes	(w501 as reference)				
In *OreR* background	−0.6191	0.5384	0.0390	−15.87	1.02×10^−56^
In *w* ^1118^ background	0.0024	1.0024	0.3705	0.06	0.952

The data come from the results presented in Figure 2.

### Dietary modification of a mitonuclear epistasis: G×G×E

The strong mitonuclear interaction for longevity with the w501 mtDNA is further modified by dietary environment. To quantify this, a series of contrasts were performed where the w501 and sm21 mtDNAs were evaluated as either two separate mitotypes or as one pooled class of mitotype, based on the fact that w501 is most closely related to the sm21 mtDNA (both are siII mtDNA haplotypes of *D. simulans* differing by only 6 nucleotides; see above). Two groups of models were evaluated (see Table 4). The first model evaluates main effects of mitotype and diet, and contrasts a model of five mitotypes (si1, sm21, w501, OreR, and Zim) vs. a four-mitotype model (si1, sm21 and w501 pooled, OreR and Zim), to test whether w501 has a significant main effect. The second set of contrasts was performed between nested models (mitotype effect nested within diet effect), and again we compared the models containing 5 mitotypes versus the reduced models of 4 mitotypes (w501 and sm21 pooled) to test whether the w501-by-diet effect is significant. These contrasts were run using data for all five diets, just the DR conditions (diets IV, II, V), or just the CR conditions (diets I, II, III).

**Table 4:** Contrasts showing the individual effect of the w501 mtDNA on longevity in two nuclear backgrounds and all diet conditions.

Main-effect models				
	*w* ^1118^		*OreR*	
Diet conditions	*χ^2^*	*P*	*χ^2^*	*P*
All 5 diets (df = 1)	0.6122 (N.S.)	0.434	289.54	6.26×10^−65^ ***
CR diets (df = 1)	0.2842 (N.S.)	0.594	169.78	1.37×10^−38^ ***
DR diets (df = 1)	20.727	5.30×10^−6^ ***	198.32	4.86×10^−45^ ***

Nested models				
	*w* ^1118^		*OreR*	
Diet conditions	*χ^2^*	*P*	*χ^2^*	*P*
All 5 diets (df = 5)	126.62	1.24×10^−25^ ***	416.70	7.45×10^−88^ ***
CR diets (df = 3)	34.279	1.73×10^−7^ ***	301.01	6.01×10^−65^ ***
DR diets (df = 3)	112.95	2.54×10^−24^ ***	201.24	2.28×10^−43^ ***

Significance codes: 0 ‘***’, 0.001 ‘**’, 0.01 ‘*’, 0.05 ‘†’.

The top half of Table 4 shows the results of the main-effect models. In the *w* ^1118^ nuclear background the sm21/w501 distinction is only significant under the DR conditions, whereas in the *OreR* nuclear background the sm21/w501 distinction significantly improves the models under all diet conditions (see Table 4, top half). These analyses confirm that the w501 effect on longevity is greater on the *OreR* background, as expected from the known epistatic interaction [29]. However, the nested models reveal that even though w501 does not have a significant main effect in the *w* ^1118^ background, the diet-by-w501 effect improves the model greatly compared to the main effect model (compare entries from top half and bottom half of Table 4). In the *OreR* background where the w501 main effect is significant, the diet-by-w501 effect greatly improves the model when all diets are analyzed, and under CR diets. Notably, in the *OreR* nuclear background under DR diets, there is little improvement in the model fit between main-effect and nested models (χ^2^ = 198.32 vs. 201.24). These analyses provide a specific example of G×G×E interactions: a particular mtDNA with distinct effects on longevity in alternative nuclear backgrounds generates unique responses to the dietary environments that modify longevity.

**Figure 3:**
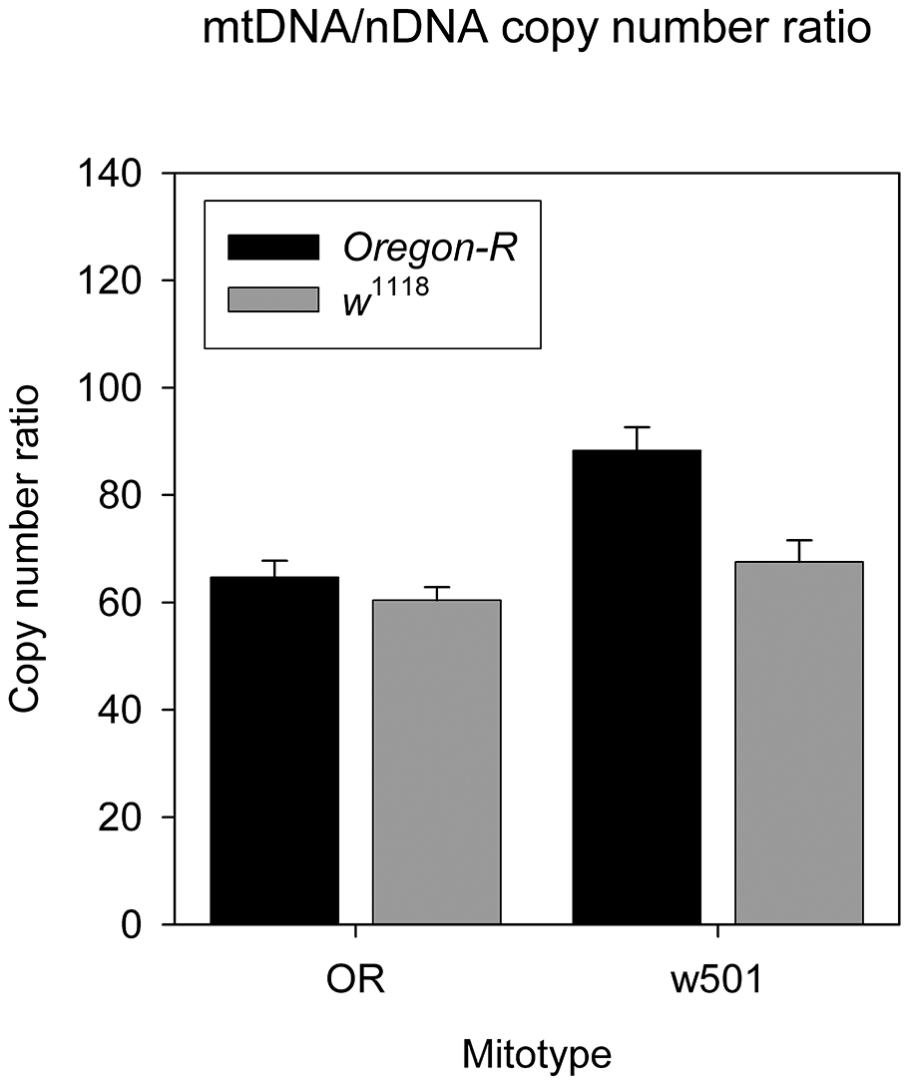
sm21 and w501 mitotypes effects on mtDNA/nDNA copy number ratio in *OreR* and *w* ^1118^ nuclear background. Error bars represent the standard error of the mean.

Mitochondrial DNA copy number also shows a w501-by-nuclear epistatic effect. We observed that the *OreR* nucleartype carrying the w501 mtDNA has a significant increase in mtDNA/nDNA copy number ratio over the sm21 mitotype and such an increase is not present in *w* ^1118^ nuclear background (Figure 3) (*F*_2,30_ = 9.79, *P* = 5.34×10^−4^, General Linear Model: mitotype effect nested within nuclear background effect). Given that mtDNA/nDNA ratio is a good indicator of mitochondrial biogenesis, this pattern may be due to the compensatory response in the presence of a w501 mito *OreR* nuclear incompatibility.

### *SIR2* overexpression, dampens the response to caloric restriction but not yeast:sugar ratio alteration

**Figure 4:**
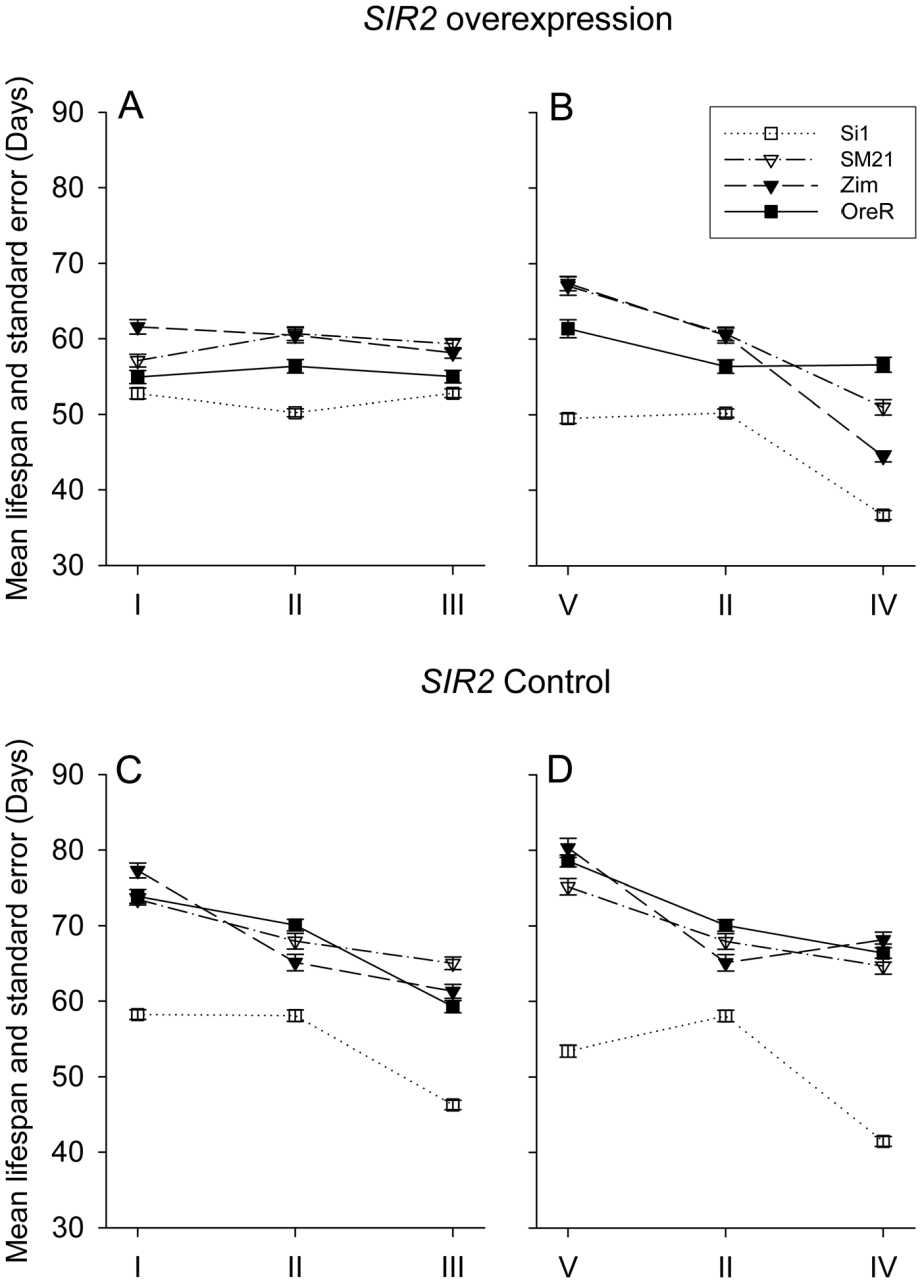
Mean lifespan and standard error of *SIR2* overexpression (A and B panels) and control (C and D panels) genotypes in different mitotype background. Mitotypes are presented with different markers and linestyles following the same scheme of figure 2. Food concentration gradient is presented in the left panels (A and C) and food composition gradients in the right panels (B and D).

Two mitotypes of each species (OreR, Zim, sm21 siI) were placed on to *SIR2* overexpression and control nuclear backgrounds to test the hypothesis of a *SIR2*-mediated mitonuclear interaction, and to examine the response of these genotypes to DR and CR dietary environments. The *SIR2* overexpression genotypes show similar mean lifespans on each of the diets of the caloric restriction gradient (diet type I, II and III) which displays a lack of CR response, while the *SIR2* control genotypes display a robust CR effect (compare the slopes of the lines in Figures 4A and 4C). The reduced response of the *SIR2* overexpression genotypes compared to the controls is consistent across different mitotypes. Statistical analyses of these data reveal that increase of caloric content increases the risk of death, however, the magnitude of increase is modulated by *SIR2* overexpression. A very strong dietary effect on survivorship is observed in the control genotypes, (Table 5, 60.1% increase in the risk of death with each diet level, *P* = 5.29×10^−103^). In the *SIR2* overexpression genotypes, this increase in risk is small but remains significant (Table 5, Caloric manipulations, hazard ratio column, 5.51% increased risk with each diet level, *P* = 0.0132).

**Table 5:** Summary of hazard ratios of *SIR2* overexpression effects and dietary effects, estimated from *SIR2* overexpression experiments with multiple diets.

		*log*(hazard ratio)	hazard ratio	S.E.M.	*z*	*P*
	Caloric manipulations (Type I, II and III)					
	*SIR2* control	*reference*				
	*SIR2* overexpression	0.6570	1.9290	0.1178	5.58	2.42×10^−8^
	dietary effect (in *SIR2* control)	0.4707	1.6011	0.2184	21.55	5.29×10^−103^
	dietary effect (in *SIR2* overexpression)	0.0536	1.0551	0.0215	2.50	0.0132
	S/Y ratio manipulations (Type V, II and IV)					
	*SIR2* control	*reference*				
	*SIR2* overexpression	0.8113	2.2509	0.1197	6.78	1.21×10^−11^
	dietary effect (in *SIR2* control)	0.4276	1.5490	0.0220	19.86	9.03×10^−88^
	dietary effect (in *SIR2* overexpression)	0.5178	1.6784	0.0231	22.45	1.28×10^−111^

Compared to the distinct responses of *SIR2* overexpression and control genotypes to the CR gradient, these same genotypes have clearly parallel responses to the DR gradient, i.e. when the animals receive dietary manipulation in a form of food composition change (sugar:yeast ratio axis, diet type IV, II and V; compare the slopes of the lines in Figures 4B to 4D). Overall, a diet rich in yeast (S/Y = 1∶3) reduces longevity whereas a diet with relatively low yeast (S/Y = 3∶1) extends longevity, relative to the balanced diet. This dietary gradient has similar effects in both *SIR2* overexpression and the control genetic background: the higher yeast diet causes a 54.9% increased risk of death in the control and 67.8% in the overexpression genotypes, *P*<0.001 for both; see Table 5, S/Y manipulations, column hazard ratio).

The observation that *SIR2* overexpression reduces the response to caloric restriction agrees well with the original report in *D. melanogaster* in which *SIR2* overexpression extended life span on high caloric diet but gains no additional extension on CR diet [40]. The new finding that *SIR2* overexpression has relatively little impact on longevity changes across a diet composition gradient, while diet V of this gradient does result in longevity extension (Figures 4B and D) suggests that the mechanisms of nutrient sensing of S/Y ratio are either independent of, or abrogate, the main effects of *SIR2*. Our results provide a clear example that CR and DR are very different processes and reveal considerable complexity underlying diet-genotype interactions that modify longevity.

### *SIR2* overexpression effects on longevity are mitotype-dependent

The plots of mean lifespan indicate that the variance among mitotypes is lower in the *SIR2* overexpression background than in the control background (Figure 4). The random effect Cox model confirms this observation. Across the CR axes (diet I II and III), the variance of the mitochondrial effect is 0.4216 in the control genotype and drops to 0.1506 with *SIR2* overexpression (Table 6). Across the DR axes (diet V, II and IV), our results show the mitotype effect to be larger than across the CR axes. Moreover, *SIR2* overexpression reduces the mitotype effect from 0.8138 to 0.3858 (Table 6). Figure 4 shows that the impact of *SIR2* overexpression is evident for mitotypes OreR, Zim and sm21, with mitotype si1 showing less response. With the si1 mitotype removed, the variance component among mitotypes diminishes (Table 6). The different responses of the two *D. simulans* mtDNAs (sm21 and si1) result in little or no impact of species-level mtDNA divergence on longevity. It is apparent that the *SIR2* overexpression reduces longevity of each mitotype on the CR and DR axes (Table 5), but the si1 mitotype is less sensitive to this effect than the other three (OreR, Zim, sm21). The distinct response of the si1 mitotype to these *SIR2* and diet experiments, and the minor role for species-level divergence, indicates that nucleotide polymorphisms between sm21 and si1 mtDNAs play a role in mediating the combined effects of *SIR2* overexpression and diet on longevity.

**Table 6:** The magnitude of mitotype effect in *SIR2* CR/DR experiments, expressed as variance components.

		w/. si1			w/o. si1	
	Nuclear Genotype	mito|_species_ *	species	replicate	mito	replicate
	Caloric manipulations (Type I, II and III)					
	*SIR2* overexpression	0.1506	0.0004	0.0018	0.0389	0.0051
	*SIR2* control	0.4216	0.0004	0.0029	0.0103	0.0058
	Y:S ratio manipulations (Type V, II and IV)					
	*SIR2* overexpression	0.3858	0.0004	0.0016	0.0356	9.11×10^−5^
	*SIR2* control	0.8138	0.1634	0.0129	0.0037	0.0138

*mitochondrial effect nested in species effect.

**Figure 5:**
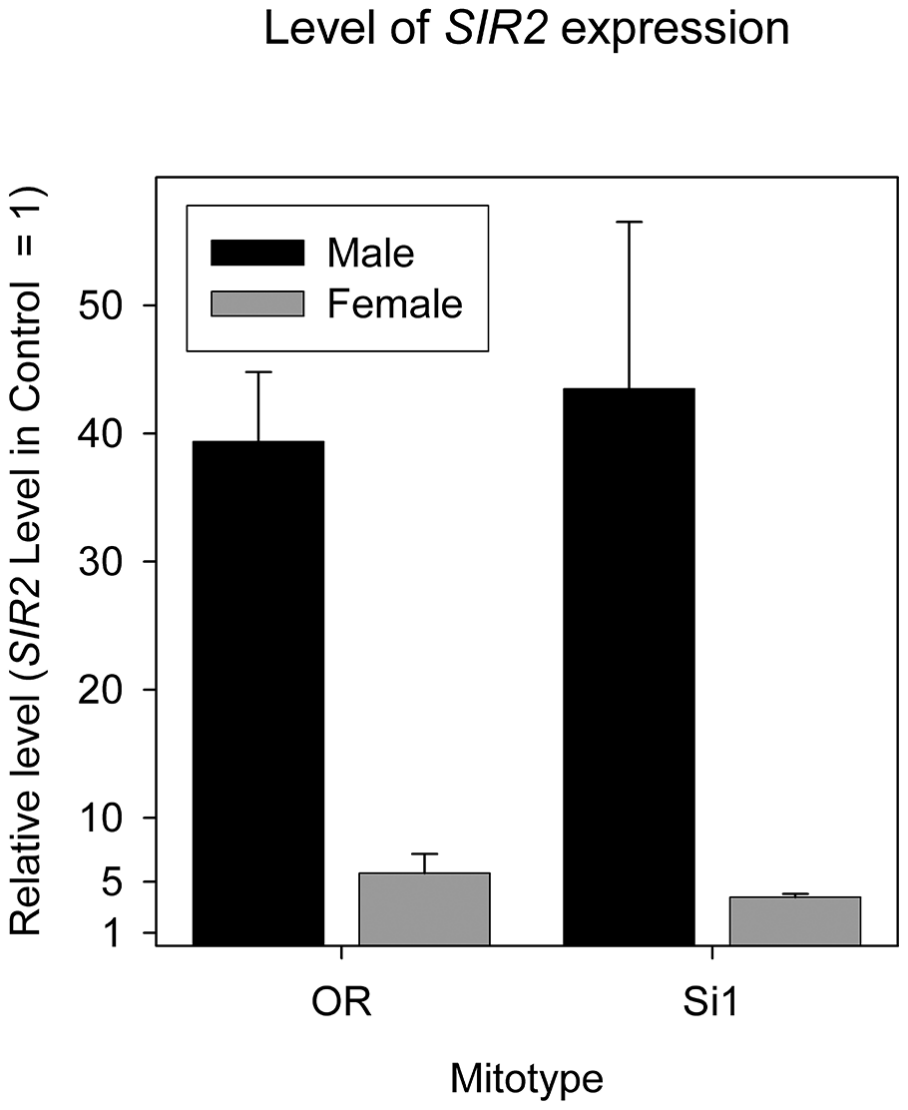
*SIR2* overexpression level in both sexes of mitotype si1 and OreR. Error bars represent the standard error of the mean of *log*(fold change); the differences are not significant in either sex (*P*>0.05 for both).

It is possible that the strong si1-*SIR2* effect is due to a very different expression level of the UAS-*SIR2* construct in the si1 mitotype background, rather than a specific mito-*SIR2* interaction. We quantified the overexpression level of UAS-*SIR2* in the siI and OreR mitotype backgrounds to test this possibility. The expression level of *SIR2* is not significantly different between siI and the most-divergent OreR (Figure 5). We note that only females were included in our demography experiment and the overexpression level is about 5× in females (Figure 5). While the 40× overexpression shown in males would likely be deleterious, we did not use males for longevity assays. Based on these results we conclude that mitotype itself does not affect the level of overexpression, indicating that the *SIR2*-siI interaction is not likely due to a spurious differential over expression mediated by mtDNA-effects on the GAL4-UAS system.

Overall, we found strong support for the effects of nuclear genotypes (*SIR2* overexpression), diet, and mitochondrial genotype on lifespan. As summarized in Table 7, all three of these terms are highly significant (*P*<0.001). We found the diet effect to be significantly altered by *SIR2* overexpression (Table 7 *χ^2^* = 233.94 *P*<0.001) on the CR axis and not significantly modified by *SIR2* on the DR axis (*χ^2^* = 3.09 *P* = 0.0793). An epistatic interaction between mitotype and *SIR2* is well supported (*χ^2^* = 77.272 and 64.074 for CR and DR respectively, *P*<0.001 for both). A mitotype-by-diet interaction is also found to be significant (*χ^2^* = 53.423 and 305.95 for CR and DR respectively, *P*<0.001 for both). As shown in the *w* ^1118^ and *OreR* experiments, we again see little support for the effect due to species level mtDNA divergence (Table 7). In summary, the results indicate that in addition to diet, mitochondrial and nuclear genotypes are important factors modulating lifespan, and that the epistatic interaction between them also has substantial effects.

**Table 7:** Mix effect Cox proportional hazard models for survivorship in *SIR2* CR/DR experiment.

		Dietary manipulations	
		Caloric	(type I, II, III)	Y:S ratio	(type V, II, IV)	
Effect	d.f.	*χ^2^*	*P*	*χ^2^*	*P*	Compare Models:
*SIR2*	1	688.14	1.13×10^−151^***	739.15	9.17×10^−163^***	
diet	1	233.94	8.24×10^−53^***	657.81	4.47×10^−145^***	Model 0 & 1
*SIR2*-diet interaction	1	136.99	1.21×10^−31^***	3.0785	0.07934 †	Model 1 & 2
mtDNA-mitotype	1	988.25	6.43×10^−217^***	1694.8	0.0***	Model 2 & 3
mtDNA-species	1	0.0094	0.9229	0.0003	0.9856	Model 3 & 4
mito- *SIR2* epistasis	1	77.272	1.49×10^−18^***	64.074	1.22×10^−15^***	Model 4 & 5a
mito-diet interaction	1	53.423	2.69×10^−13^***	305.95	1.67×10^−68^***	Model 4 & 5b
replicate	1	0.0072	0.9325	0.0102	0.9196	Model 5a & 6

Significance codes: 0 ‘***’ 0.001 ‘**’ 0.01 ‘*’ 0.05 ‘†’.

Model 0: *SIR2 (Note 1).*

Model 1: *SIR2* + diet *(Note 2).*

Model 2: *SIR2* + diet + *SIR2* |_diet_.

Model 3: *SIR2* + diet + *SIR2*|_diet_ + mito.

Model 4: *SIR2* + diet + *SIR2*|_diet_ + mito|_species_+ species.

Model 5a: *SIR2* + diet + *SIR2* |_diet_ + *SIR2*|(mito|_species_ + species) + mito|_species_ + species.

Model 5b: *SIR2* + diet + *SIR2* |_diet_ + diet|(mito|_species_ + species) + mito|_species_ + species.

Model 6: *SIR2* + diet + *SIR2* |_diet_ + *SIR2*|(mito|_species_ + species) + mito|_species_ + species + replicate.

Note 1: fixed effect, such as diet, is show with underline.

Note 2: diet is modeled as an ordinal variable rather than a nominal variable.

## Discussion

In this study we have quantified the effects of mitochondrial genotypes, nuclear genotypes and dietary modifications on the lifespan of *Drosophila*. Our goal was to provide a comprehensive survey of the range of effects across each of these interacting genetic and dietary factors. No previous study has examined the complexity of interactions among these factors in an effort to sort out main effects, epistatic effects and genotype-by-environment interactions.

Our results show that the main effects of variation in mtDNA, nuclear genetic background and diet can be significantly modified by the interaction between each factor, using controlled orthogonal designs capable of quantify interaction effects. Among mtDNAs, there are mitotypes that show parallel shifts in lifespan across diets or nuclear backgrounds, but there are others that show very different responses with crossing reactions norms among diets (Figures 2 and 4). For nuclear backgrounds, there are distinct responses to diet for each of the four genotypes studied (*w* ^1118^ and *OreR*, *SIR2* overexpression and control). And for caloric or dietary restriction, the efficacy of this manipulation is highly dependent on genotype, both nuclear and mitochondrial. Overall, our results reveal that the interactions are arguably more important than the main effects alone for *Drosophila* life span. The interactions are not simply pair-wise, as mito-nuclear interactions are modified by diet. These genotype-by-genotype-by-environment, or G×G×E (technically Mito × Nuclear × Diet) effects raise the question of how best to proceed with the identification of general effects that modify longevity.

### MtDNA mutations, mitonuclear epistasis and purifying selection

A general result regarding mtDNA effects on longevity is the different impacts of point mutations versus deeper mtDNA divergence. The high mutation rate of mtDNA results in high levels of mtDNA variation in most animals, including humans. These new mutants are actively tested for function with nuclear genetic variation across each generation, and chance combinations of nuclear and mitochondrial alleles can generate novel phenotypes (epistatic interactions) that are not predicted from the main effects of either genome. The large number of nuclear-encoded subunits of the mitochondrial electron transport chain and mitochondrial translation machinery make a particularly large mutational target for novel variants that must interact with the mtDNA encoded subunits that contribute to these critical cellular processes.

One result of particular interest is the effect of the tRNA^Tyr^ mutation in w501 mtDNA, which reduces lifespan on the *OreR* nuclear background but can show average longevity on the *w^1118^* background. This demonstrates a particular case of 2-nucleotide mitonuclear incompatibility due to mis-match between mitochondrial- and nuclear-encoded components. In this particular case of w501 mtDNA, the *OreR* and *w* ^1118^ nuclear backgrounds carry different tRNA-synthetase alleles that functionally interact with the w501 tRNA^Tyr^ mutation. Although, admittedly, a *D. simulans* mitochondrial-*D. melanogaster* nuclear genome combination is unlikely in nature, mitochondrial dysfunction due to tRNA mutation is not at all rare. Notably, exercise intolerance in humans results from a mutation in this same mt-tRNA^Tyr^ gene [41], [42], [43]. Indeed, mitochondrial tRNA diseases involving similar mitochondrial-nuclear dysfunction in human are common, such as MERRF syndrome (Myoclonic Epilepsy with Ragged Red Fibers) [44], which is caused by mutation in mitochondrial tRNA^Lys^. Therefore, the principle of mitonuclear epistatic interactions is a general problem that has received little attention until recently [28], [29], [30].

One rather unexpected finding is that the w501- *OreR* interaction appears to result in small, yet significant, upregulation of mitochondrial copy number. The regulatory mechanism of steady level of mtDNA copy number is thought to be a complex network consisting of both mitochondrial and nuclear encoded components, with many details yet to be elucidated. The major nuclear encoded components includes mitochondrial transcription factor A (TFAM), mitochondrial DNA polymerase γ (PLOG), mitochondrial single strand binding protein (mtSSB) [45]. The sequence of the mtDNA in the origin of replication region of the D-loop, presumably also plays an important role affecting mtDNA level [46]. Our result suggests that mitochondrial translational deficiency, such as the one leading to the w501- *OreR* interaction, may also be a part in the mtDNA level regulatory network, possibly acting though a compensatory mechanism.

In both the wild type nuclear backgrounds, *w* ^1118^ and *OreR*, we demonstrated that dietary restriction effect is highly dependent on mitotype (Table 2). These genome-wide patterns provide strong motivation to test the hypothesis that epistasis also exists between mitotypes and genetic pathways known to influence dietary restriction. We tested this hypothesis using a mito-*SIR2* interaction. The up-regulation of *SIR2* leads to lifespan extension in many model organisms [15], [18], [40], [47], [48], [49] and there are sound reasons why this process may involve a mito-*SIR2* interaction. First, *SIR2* is an NAD^+^ consuming deacetylase [50], [51] and mitochondria oxidize NADH to NAD^+^ during oxidative phosphorylation by NADH dehydrogenase (Complex I). Interaction between *SIR2* and mitochondria can stem from this sharing of the same cofactor pool. Second, *SIR2*-mito interactions may also arise through the upregulation of mitochondrial function by mimicking DR, which also involves other members of the sirtuin family [27], [52]. This pattern of epistatic interaction is consistent with a number of studies showing that mitochondrial function is enhanced, not only by sirtuins, but also other pathways that mimic DR [10], [14], [22], [23], [34], [53].

Due to the sequence divergence of the mitotypes included in this study, different mitotypes are expected to cause different levels of mitonuclear incompatibility, some mild while others may be severe. To the extent that overexpressing *SIR2* mimics DR and enhances mitochondrial function, the distinct set of mtDNA haplotypes used in our study has the potential to identify novel functional interactions between *SIR2* and mitochondria. Our initial expectation was that the divergent *D. simulans* mtDNA would generate higher levels of incompatibility and show greater differences in longevity. However, it was a particular mitotype, not the species-level divergence that is most informative (Figure 4, siI mitotype). Further genetic mapping and biochemical analyses are required to understand how siI mitotype mediates a unique response to *SIR2*, but the mutations that are unique to the siI mitotype provide a list of candidates that could guide these studies (see Table S1).

Our results have consistently falsified the hypothesis that divergent mtDNAs from a different species represent ‘strongly incompatible’ mtDNA genotypes. In most nuclear backgrounds, and across most diet treatments, our data show that the species-level divergence of mtDNAs (∼100 amino acids, see supplementary Table S1) has little phenotypic effect, while individual mtDNAs can have pronounced effects (e.g., w501 and siI mtDNAs; Figures 2 and 4, Tables 2 and 7). If purifying selection has been a general force during the divergence of mtDNAs, the large sequence divergence between species may be accompanied by little accumulated functional divergence. Likewise, the nature of purifying selection will determine the mutation-selection balance that permits individual mutant mitotypes carrying deleterious mutations to persist in populations, with measurable effects on longevity and other performance traits. These results suggests that additional screens of very closely related mtDNA may uncover informative SNPs showing strong epistatic interactions with nuclear factors affecting longevity.

### Distinct mitonuclear mechanisms for dietary and caloric restriction

Our results also imply that the responses to food concentration or composition changes involve different mechanisms. Altering caloric content and changing yeast concentration are currently two main approaches of caloric vs. dietary restriction (CR and DR, respectively) in *D. melanogaster*. The former changes diet concentration while keeping sugar/yeast ratio constant [37], [54]. The latter approach can alter the sugar/yeast ratio while keeping total calories constant [37], or restrict the amount of yeast, resulting in both sugar/yeast ratio and total caloric changes [55]. We found that the *SIR2* control genotype shows a strong response to CR, but *SIR2* overexpression does not respond to CR, and lifespan changes little on different diets as long as yeast:sugar ratio is unchanged (compare Figure 4A to 4C). In contrast, the lifespan of *SIR2* overexpression still displays response to isocaloric DR involving sugar/yeast ratio changes (compare Figure 4B to 4D). These observations collectively suggest that *SIR2* is involved in sensing food concentration changes but has less of a role in sensing composition changes or yeast:sugar ratio. Thus, our results are consistent with the hypothesis that *SIR2* regulates lifespan, but clearly show that *SIR2*'s manner of longevity regulation is dependent on diet, which is indeed a case of genotype × diet interaction. A possible model to explain this result is that *SIR2* is only involved in CR but not DR. When *SIR2* is already overexpressed, additional CR can't result in further extension lifespan. However under DR, which may be *SIR2* independent, this dietary modification remains effective regardless of *SIR2* level. Therefore in both control and *SIR2* overexpression condition, DR results in similar extension of lifespan.

In contrast to other reports, we found overexpression of *SIR2* overall decreases life span. *SIR2* is an important gene in aging research as it has suggested a link between metabolism and longevity [16], [56]. A polyphenol resveratrol, found in red wine, was suggested to be a *SIR2* activator and has been demonstrated to lead to variety of benefits that are associated with DR [18], [47]. However, the roles of both *SIR2* and resveratrol have been challenged [57], [58], [59]. Absence of life span extension by *SIR2* overexpression has also been reported before [60], [61], [62], [63]. In our case, we suspect the lifespan decrease we observe is due to the expression level of *SIR2* that is not optimal for longevity extension or related to developmental effects in larval life. Here we found the expression of *SIR2* driven by a strong ubiquitous da-GAL4 driver to be about 5 times that of controls in females and 40 times that of controls in males (we used females for all longevity experiments). Previously, the similar ∼5× over-expression of *SIR2* has been shown, in two studies, to increase or have no effect on longevity in *Drosophila* [61], [64]. It is known that *SIR2* expression is high in the embryo stage but moderate in adult with high expression in brain, salivary gland, female ovary and spermatheca [65], [66], displaying a highly tissue specific pattern. Therefore, the absence of lifespan extension in our experiments is also possibly due to *SIR2* expression in tissues and stages where overexpression can become detrimental. We note that one goal of this study was to identify genetic interactions between *SIR2* and mtDNA variants as they affect the response to DR and CR, and the si1 mtDNA-*SIR2* interaction is a promising result. However, we acknowledge further studies using stage- and tissue-specific *SIR2* overexpression with moderate dosage are needed to resolve the details of *SIR2*-mtDNA genetic interactions.

Aging is a complex process and extending life span requires the coordination of a large number of genes, pathways, and environments, among which dietary regime is probably specially important. The interaction between them is an intrinsic nature of the aging process. These interactions may often appear as annoying sources of experimental complication that reduces the repeatability in particular experiments. For example, different mouse genotypes respond very differently to the same dietary environment [36], [21], which is essentially a form of G×E interaction. Moreover, alternative combinations of alleles from different loci can lead to different phenotypic response [40], [61], which is in fact an epistatic or G×G interaction. Both G×E and G×G interactions have been demonstrated and are beginning to draw attention in aging research. However, the greater challenge, as we have elucidated in this work, is a more general G×G×E interaction. That is, the effects of mutations that affect lifespan are heavily dependent on the interaction between genetic background and dietary regime simultaneously. If a goal in aging research is to understand the complexity of factors that may help ensure increased healthspan in humans, we must confront the complexity of interactions presented by genetically variable humans living in the variety of dietary environments so common today. Here we have sought to provide an example of such an effort in a model organism where we can begin to manipulate these factors and eventually elucidate the mechanisms of the interactions that have undoubtedly been important throughout evolution.

## Materials and Methods

### Strains and mitonuclear genotypes

Longevity analyses were carried out on lines of *D. melanogaster* carrying alternative mtDNAs from *D. melanogaster* and its sister species *D. simulans*. We generated three sets of lines on each of three different nuclear genetic backgrounds, *Oregon R* (*OreR*), *w* ^1118^ (Bloomington stock number 6326) and daughterless-GAL4 (da-GAL4, Bloomington stock number 5460), each carrying alternative mtDNAs. The *D. melanogaster* mitochondria are from the Oregon R (OreR) strain and Zim53 (Zim), from Zimbabwe [67], [68]. Also included are *Drosophila simulans* mitochondrial haplotypes si1 from Hawaii, sm21 from strain C167.4 (*Drosophila* Genetic Resource Center stock number 107850), and w501, from strain white501 (w501, *Drosophila* Species Stock Center stock number 14021-0251.195). Introgression of *D. simulans* mitochondria into flies carrying *D. melanogaster* nuclear genome is made possible by a rescue cross involving *D. simulans* strain C167.4 as females and *D. melanogaster* strain *In(1A)B* as males [28], [39], followed by balancer chromosome replacement to ensure the mito-replaced strains have the same set of *D. melanogaster* nuclear chromosomes of the original strain (Supplementary Figure S1). For simplicity, we will refer to different mitochondrial haplotypes as “mitotypes” (*cf. mito*chondrial geno*types*) and nuclear chromosomal composition as “nucleartype”. When contrasting mitotypes in different nucleartypes, the following terminology will be used: ‘the OreR mitotype has different longevity on alternative nuclear backgrounds’; or the *w* ^1118^ nucleartype shows different longevities on different mtDNA backgrounds'. We use the term “genotype” or “mitonuclear genotype” to refer to the combination of mtDNA and nuclear chromosomes that are constructed in a particular genetic line, using the notation: mtDNA; nuclearDNA. For example, the mitonuclear genotype w501; *w* ^1118^ carries the *D. simulans* w501 mtDNA and the *D. melanogaster w* ^1118^ nuclear chromosomes.

The mitotype panel covers a wide range of mitochondrial mutations: the OreR mitotype differs from the *D. melanogaster* Zim mitotype by 18 amino acid substitutions, and by 103 amino acid substitutions from the most diverged mitotype, *D. simulans* si1. The mitotypes are all fully sequenced and details of the molecular evolutionary divergence and the phylogenetic relationships can be found in Ballard [68], and Montooth et al.[39]. The Genebank sequence accession numbers for the sequences of mitotypes are: OreR AF200828, Zim AF200829, siI AF200835, sm21 KC244283 and w501 KC244284.

Overexpression of *SIR2* was achieved by driving the expression of UAS-*SIR2* via a ubiquitous daughterless-GAL4 driver. UAS-*SIR2* construct was kindly provided by Drs. Jason Wood and Stephen Helfand. Each mitotype described above was introduced into the da-GAL4 construct using balancer chromosome substitution. *SIR2* overexpression in different mitotype backgrounds is made possible by the mito-replaced da-GAL4 strains. Overexpression genotypes were obtained by crossing UAS-*SIR2* males to mitotype-replaced da-GAL4 females. The control genotypes are generated by crossing the *w* ^-^ strain, in which UAS-*SIR2* is constructed, to da-GAL4 females. To keep UAS-*SIR2* and its control *w* ^-^ strain (+) has the same genetic background, they were maintained as UAS-*SIR2*/+ heterozygote and sorted by eye color to get UAS-*SIR2*/UAS-*SIR2* and +/+ males to minimizing the chance of possible fixed genetic divergence that may arise between the *SIR2* and the + genotypes if both were to be maintained as individual homozygote strains. Additional control genotypes, such as a control for the GAL4 line, would have been informative. However, the primary goal is to determine the reality (and magnitude) of interaction effects. Therefore, the alternative design is not employed. In our approach the over-expression genotype (da-GAL4/UAS-SIR2) and the non-overexpression genotype (da-GAL/+) both carry the same whole genome heterozygous background (da-GAL4 background/UAS-*SIR2* background). Therefore the whole genome heterozygous background will minimize the effect of any fixed mutations on either the da-GAL4 background or the UAS-*SIR2* background, and will reveal a more general picture how *SIR2* and mtDNA will interact in any randomly chosen genetic background from a wild population.

### Dietary manipulations

A five-diet design was used to study the effects of both food composition and food concentration on life span, termed diet I, II, II, IV, V (see Figure 1). The ingredients of the five diets (all in grams per liter); type I: 50 sucrose + 50 yeast, type II: 100 sucrose + 100 yeast, type III: 150 sucrose + 150 yeast, type IV: 50 sucrose + 150 yeast and type V: 150 sucrose + 50 yeast. All of these are prepared with 15 g/dl agar and supplemented with 0.2 g/dl Tegosept as fungal suppressor. The food making procedure follows the protocol described in [35]. Briefly, ingredients are well mixed in hot water and then autoclaved at liquid cycle (120°C for 25 min) to achieve sterility and uniform cooking conditions. The food mix is then allowed to cool down before addition of Tegosept using 20 g/dl stock solution. Note that type I, II and III are exactly the same as 0.5 N, 1.0 N and 1.5 N food used by other groups [35], [36], [40]. Type I, II and III form a concentration gradient where the yeast sugar ratio is constant and the total concentration and caloric content varies. Mair et al. reported very similar caloric contents for carbohydrate and autolysed yeast powder, 4.0 and 4.02 kcal/g respectively [37]. Therefore, type V, II and IV form a composition gradient where the caloric content is approximately constant but the food composition (sugar/yeast ratio) varies. “Standard food” is the common cornmeal food for general stock keeping and is made of 10 g/l agar, 100 g/l sucrose, 50 g/l cornmeal and 50 g/l cornmeal yeast.

### Demography

Fly lifespan was measured by counting individual deaths in demography cages. Briefly, newly eclosed flies were collected over a 24 hour period, held on regular food for 4 days as mixed sex adults, and then sorted by sex. Demography experiments were only conducted on females. 100 females were placed in 1 liter cages in triplicate and were supplied with different types of food. On Mondays, Wednesdays and Fridays, food was renewed and death recorded. Cages were kept in climate controlled chambers maintaining a 12/12 hours light/dark cycle at 25°C.

### Molecular assays

Quantification of the ratio of mitochondrial to nuclear DNA (mtDNA/nDNA) and *SIR2* expression level were carried out by a quantitative PCR (qPCR) based method. To avoid amplification bias due to relative high A/T richness of mtDNA compared to genomic DNA we chose a G/C rich region in mtDNA to make mtDNA and nDNA primer sets comparable. Both mtDNA primers and nDNA primers were aligned against available sequence data in the NCBI database to ensure they map to highly conserved regions and are not likely to be affected by mismatch due to sequence polymorphism. We followed the DNA extraction method suggested by Guo et.al [69] to avoid underestimating the abundance of mitochondrial DNA. The primer sequences are as follows: mtDNA: 5′-GATTAGCTACTTTACATGGAACTC-3′ and 5′-CTGCTATAATAGCAAATACAGCTC-3′ adjacent to the mitochondrial genomic region of cytochrome c oxidase subunit I gene; nDNA: 5′-AACTCTGCTGCTACTTATCG-3′ and 5′-CAGGATCAGGATGGAATAGTATC-3′ adjacent to the nuclear region of NADH dehydrogenase 51 kDa subunit gene.

*SIR2* expression level is quantified using ddCT method with a pair of primers within *SIR2*: 5′-TCATGAAGCCGGATATCGT-3′ and 5′-GGTATGCTGCTGGGAATG-3′ together with the reference primers in house keeping gene *GAPDH*: 5′-CCACTGCCGAGGAGGTCAACTAC-3′ and 5′-CATGCTCAGGGTGATTGCGTATGC-3′. Both qPCR experiments are done with 10-day-old whole flies reared on regular food. mtDNA/nDNA was measured in males only while *SIR2* expression levels were assayed in both males and females. For both experiments, 3 biological replicates were assayed with 3 technical replicates.

### Statistical analyses

To test the effect of mitotype, nuclear genotype and diet on lifespan, we analyzed the demography data using a mixed-effect Cox proportional hazard model. The mixed-effect Cox model is an extension to the popular Cox proportional hazard model [70] to include random effects. The Cox model estimates the *log*(hazard ratios) from survivorship of different groups. Two groups having a hazard ratio equal to 1 (or equivalently *log*(hazard) equal to 0) suggests the groups are equally likely to die. Similarly, negative *log*(hazard) or positive *log*(hazard) suggests one group has higher or lower probability to die than the other group and subsequently survives shorter or longer, respectively. In a mixed-effect Cox model, random effects are included. Random effects are modeled assuming their *log*(hazard ratio) are drawn from Gaussian distributions with zero mean and unknown variances. The unknown Gaussian variances are numerically solved by finding the maximum of integrated partial likelihood using an iterative method [71]. The model was originally developed for clinical application. For example, a type of drug is tested in multiple hospitals in order to determine its ability to extend patient survival (by comparing treatment group survival to control group in each hospital). However, it is also important to know the amount of variation among hospitals especially how big the variation is in relation to the treatment effect. The among-hospital variation is considered as a random effect as it estimates the variation among *all* hospitals represented by the hospitals sampled. The treatment effect is, on the contrary, considered as a fixed effect. The mixed-effect Cox model enabled us to examine both fixed terms (such as diet type analogous to drug effect in this example) and random terms (such as genetic background effect and replicate noise, analogous to variation among hospitals) simultaneously. It also made examining variance components possible such that we can determine the relative magnitude of different random effects. Therefore their relative importance can be evaluated. In more practical terms, variance components estimated by the mixed-effect Cox proportional hazard model is a metric of the magnitude of effects, with a larger value indicating an effect is substantial and a small value indicating an effect is negligible. These tests are not possible with a classic Cox proportional hazard model with support for only fixed effects. The mixed effect Cox model is implemented using R package *coxme()* (http://cran.r-project.org/web/packages/coxme/). Additionally, the significance tests of overall contributions of each term are conducted by likelihood ratio test by comparing full models versus reduced models. Log-likelihood ratio tests are implemented using R Analysis of Deviance package *anova()* (http://stat.ethz.ch/R-manual/R-patched/library/stats/html/anova.glm.html).

## Supporting Information

Figure S1 Schematics for mitochondrial replacement by balancer substitution.
(PDF)

Table S1 Amino Acid polymorphisms among the mitotypes. "." indicates indentical sequence to the reference (Zim53).
(PDF)
