# Supplementary figures and images for: G×G×E for Lifespan in Drosophila: Mitochondrial, Nuclear, and Dietary Interactions that Modify Longevity
Source: PLoS Genet. 2014 May 15;10(5):e1004354. doi: 10.1371/journal.pgen.1004354 (PMC4022469; doi:10.1371/journal.pgen.1004354)

Figure S1, Schematics for mitochondrial replacement by balancer substitution.

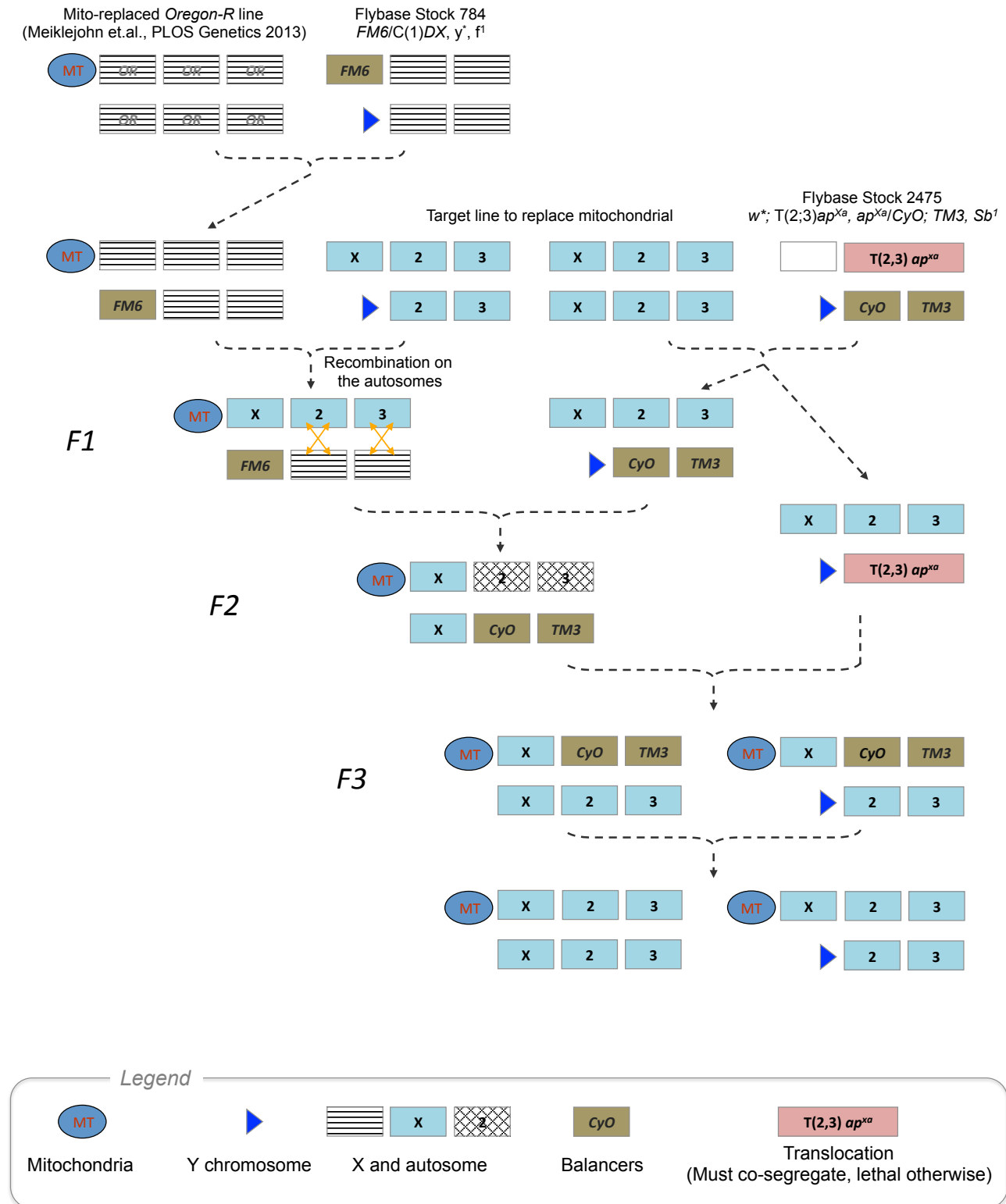

Supplement: Figure S1 — Schematics for mitochondrial replacement by balancer substitution. (PDF) [file pgen.1004354.s001.pdf]
