# Supplementary material for: G×G×E for Lifespan in Drosophila: Mitochondrial, Nuclear, and Dietary Interactions that Modify Longevity
Source: PLoS Genet. 2014 May 15;10(5):e1004354. doi: 10.1371/journal.pgen.1004354 (PMC4022469; doi:10.1371/journal.pgen.1004354)
Supplement: Table S1 — Amino Acid polymorphisms among the mitotypes. "." indicates indentical sequence to the reference (Zim53). (PDF) [file pgen.1004354.s002.pdf]

Table S1. Amino Acid polymorphisms among the mitotypes. "." indicates indentical sequence to the reference (Zim53).

			1									10										20										30										40
		Gene:	*ND2*															*COX1*					*COX2*						*ATP8*			*ATP6*										
		Position:	65	84	142	148	192	196	198	274	276	277	313	315	317	321	333	1	127	411	450	468	115	129	130	143	165	218	26	32	34	17	28	45	115	119	137	177	180	185	187	192
D.melanogaster	Zim53		V	M	S	F	I	I	L	M	T	L	N	I	Y	M	L	S	F	H	I	F	N	M	T	V	V	Y	I	M	N	F	L	M	L	I	I	N	P	I	V	M
	OreR		.	.	.	Y	.	.	.	.	.	I	.	.	.	.	.	.	Y	.	.	.	.	.	.	.	.	.	.	.	.	.	.	.	.	.	.	K	S	M	M	.
D.simulans	siII	(SM21 & w501)	A	L	T	Y	.	V	F	L	L	M	I	S	T	L	M	P	Y	Q	V	Y	.	S	I	I	I	H	.	.	D	L	.	V	.	F	.	.	.	L	.	T
	siI		A	L	.	Y	F	.	.	L	.	.	I	N	T	L	M	P	Y	Q	.	.	S	T	.	I	.	H	M	I	.	L	I	V	M	.	V	.	.	L	I	T

	41									50										60										70										80
	COX3								*ND3*			*ND5*																											*ND4*	
	41	45	92	170	172	176	193	199	20	29	81	7	8	12	27	36	42	65	77	81	149	152	179	184	244	289	422	462	466	470	476	500	516	539	557	567	568	569	9	12
Zim53	I	V	V	M	I	I	I	F	F	A	M	V	N	M	D	L	M	S	M	H	L	S	M	V	M	L	M	V	F	I	F	I	V	M	L	L	-	I	L	I
OreR	.	.	.	L	.	.	.	.	.	.	.	.	.	.	.	.	.	.	.	.	.	.	.	.	.	.	.	.	.	.	.	M	.	.	.	.	.	.	.	.
siII	M	L	.	L	V	M	V	Y	I	.	.	I	Y	I	N	V	S	A	S	N	F	A	V	M	L	F	.	.	S	.	Y	.	I	T	M	M	N	F	V	S
siI	M	L	I	L	V	M	V	Y	I	G	I	I	Y	I	N	V	S	A	S	N	F	A	.	M	L	.	L	M	.	V	Y	.	I	T	M	M	N	F	.	T

	81									90										100										110				
															ND4L		*ND6*											*Cytb*		*ND1*				
	14	17	29	37	78	82	94	141	231	236	298	383	386	426	57	79	39	82	86	90	93	96	100	101	102	107	159	109	357	170	186	190	191	270
Zim53	F	I	F	L	M	H	I	L	M	S	C	L	F	L	S	V	L	M	L	L	I	L	F	I	M	S	I	K	V	F	A	M	S	V
OreR	.	.	.	.	.	.	.	.	.	.	.	.	.	.	.	.	.	.	.	.	.	.	.	.	.	.	.	.	.	.	.	V	.	.
siII	L	.	.	.	S	Y	V	V	.	A	S	M	L	F	N	.	M	I	V	F	.	.	.	.	I	.	V	M	.	.	G	L	T	I
siI	L	M	V	V	S	Y	V	V	L	A	S	M	L	F	N	I	M	I	V	.	L	M	L	V	I	F	V	M	I	Y	.	L	T	A
